# Pseudophakic cystoid macular oedema and posterior capsular opacification rates after combined phaco‐trabeculectomy vs. phaco alone

**DOI:** 10.1111/aos.16766

**Published:** 2024-10-11

**Authors:** Eliya Levinger, Michael Ostrovsky, Asaf Friehmann, Omar Elhaddad, Derek Tole, Kieren Darcy, Duncan Leadbetter, Raimo Tuuminen, Mordechai Goldberg, Asaf Achiron

**Affiliations:** ^1^ Tel Aviv Sourasky Medical Center Tel Aviv Israel; ^2^ School of Medicine Tel Aviv University Tel Aviv Israel; ^3^ Ophthalmology Department Meir Medical Center Kfar Saba Israel; ^4^ University Hospitals Bristol and Weston NHS Foundation Trust Bristol Eye Hospital Bristol UK; ^5^ Faculty of Medicine Alexandria University Alexandria Egypt; ^6^ Sulis Hospital Bath UK; ^7^ Department of Ophthalmology Kymenlaakso Central Hospital Kotka Finland; ^8^ Helsinki Retina Research Group, Faculty of Medicine University of Helsinki Helsinki Finland; ^9^ Glaucoma Service, Ophthalmology Department Shaare Zedek Medical Center Jerusalem Israel

**Keywords:** cataract surgery, posterior capsular opacification, pseudophakic cystoid macular oedema, trabeculectomy

## Abstract

**Purpose:**

To assess the risk for pseudophakic cystoid macular oedema (PCME) and posterior capsular opacification (PCO) associated with combined cataract surgery and trabeculectomy compared to cataract surgery alone.

**Methods:**

Data analysis of subjects who underwent routine cataract surgery without and with concomitant trabeculectomy at the Department of Ophthalmology, Bristol Eye Hospital, the UK, between January 2008 and December 2017. Odds ratios (ORs) for PCME between the types of surgeries were calculated using univariate and multivariate regression analysis. Multivariate Cox regression controlling for age and gender was used to estimate the hazard ratio (HR) for neodymium‐doped yttrium aluminium garnet (Nd:YAG) laser capsulotomies.

**Results:**

This study included 56 973 cataract surgeries without and 288 with concomitant trabeculectomy (phaco‐trab) with a mean follow‐up time of 6.9 ± 4.2 years. Baseline variables (age and gender, diabetes, pseudoexfoliation, use of pupil expansion device and postoperative follow‐up time) were comparable between the groups. Postoperative rates of PCME remained non‐significant between the cataract surgery and phaco‐trabe groups both in uni‐ and multi‐variate analysis (OR 0.347, 95%CI 0.049–2.477, *p* = 0.291). Furthermore, in Cox regression analysis adjusted for the patients' age and gender, Nd:YAG laser capsulotomy rates remained non‐significant between the cataract surgery and phaco‐trabe groups (HR 1.250, 95%CI 0.883–1.769, *p* = 0.209).

**Conclusions:**

In our large cohort study, combining trabeculectomy with cataract surgery did not predispose to an increased PCME or Nd:YAG laser capsulotomy rates.

## 1 INTRODUCTION

Glaucoma is the world's leading cause of irreversible blindness, with over 110 million people projected to suffer from glaucoma by 2040 (Tham et al., 2014). Trabeculectomy is traditionally considered the gold standard surgical procedure for managing glaucoma and the operation of choice for glaucoma refractory to medical and laser treatment or when the target IOP is very low (Lim, 2022; Vinod et al., 2017). However, a growing body of evidence shows cataract progression following glaucoma surgery (Hylton et al., 2003; Zhang et al., 2015). Combining trabeculectomy with cataract surgery, compared to cataract surgery alone, was deeply investigated, mainly regarding its effect on intraocular pressure (IOP), late visual field changes and early postoperative complications (Vizzeri & Weinreb, 2010). However, only a few publications compared posterior capsular opacification (PCO) and pseudophakic cystoid macular (PCME), Table S1 (Ghadamzadeh et al., 2022; Hansapinyo et al., 2020; Paul et al., 2014; Shin et al., 2002; Tham et al., 2010; Ventura‐Abreu et al., 2021). Therefore, in this study, we compared the risk of PCO and PCME in phacoemulsification cataract extraction with and without concomitant trabeculectomy in a large cohort of patients.

## 2 MATERIALS

This was a registry‐based retrospective cohort study of consecutive adult cataract surgeries performed at the Department of Ophthalmology, Bristol Eye Hospital, UK. Patients were enrolled between January 2008 and December 2017 and admitted according to the national cataract management guidelines. This study received the local ethics community approval (CORN/SE/2021‐2022/02 and was presented to the local audit authority) and adhered to the tenets of the Declaration of Helsinki. Inclusion criteria were all adult patients who underwent phacoemulsification surgery and intraocular lens implantation with or without trabeculectomy.

### 2.1 Data acquisition and subjects

Data were collected from the Medisoft electronic medical record (EMR) system (Medisoft, Ltd., Leeds, UK) (Ting et al., 2023). Clinical variables were registered for age at surgery and gender (male/female), date of cataract surgery and laterality, DM status (no/yes), the existence of pseudoexfoliation syndrome (PXF) (no/yes), concomitant trabeculectomy (no/yes), and Nd:YAG laser capsulotomy after the surgery. PCME was based on clinical judgement (postoperative intraretinal fluid in the optical coherence tomography scans together with the deterioration of best‐corrected visual acuity gain from that of expected). Cases were detected by the Medisoft EMR system structured report, defined by the diagnosis box of PCME (no/yes), without OCT verification. All surgeries for patients under 18 years were excluded (*N* = 105). Follow‐up was calculated as the mean time from surgery to the last point of clinical outcome (date of new‐onset PCME or Nd:YAG laser capsulotomy).

### 2.2 Statistical analyses

Data are presented as mean ± standard deviation (SD) or absolute values and proportions. Analysis was performed using IBM SPSS Statistics 27 (IBM SPSS Statistics for Windows, Version 27.0. IBM Corp., Armonk, NY). A two‐factor Chi‐square test was used for qualitative data and a Student's *t*‐test for continuous variables. Odds ratios (ORs) for PCME were calculated using multivariate regression, with age and gender as covariates. Kaplan–Meier curves were generated, and a log‐rank test was used to assess Nd:YAG laser capsulotomy‐free survival. Multivariate Cox regression controlling for age and gender was used to estimate the hazard ratio (HR) for Nd:YAG laser capsulotomies. *p*‐values less than 0.05 were considered statistically significant.

## 3 RESULTS

Included were 57 261 consecutive cataract surgeries. Overall, 56 973 eyes were operated without, and 288 were operated with concomitant trabeculectomy. Baseline variables regarding the mean age of patients, gender distribution, eye laterality, diabetes, PXF, use of a pupil expansion device and follow‐up time after the surgery were comparable between the study groups (Table 1).

**TABLE 1:** Baseline parameters according to the use of pupil expansion device.

	Phaco alone *N* = 56 973	Combined phaco‐trab *N* = 288	*p*‐Value
Age (years)	79.2 ± 11.2	78.3 ± 14.2	0.156
Gender (male:female)	23 350:33623 (41.0:59.0%)	128:160 (44.4:55.6%)	0.234
Laterality (right:left)	29 123:27850 (51.1:48.9%)	135:160 (46.9:53.1%)	0.151
DM^a^	10 785 (20.2%)	35 (16.0%)	0.117
PXF^b^	107 (0.19%)	1 (0.36%)	0.514
Pupil expansion device	851 (1.49%)	4 (1.39%)	0.884
Follow‐up time (years)	6.9 ± 4.2	6.5 ± 4.0	0.092

*Note*: Data are given as mean (±SD) or absolute values (and proportions). For two‐group comparisons, two‐factor Chi‐squared test was used for qualitative data and Student's *t*‐test for continuous variables. Data missing ^a^ *N* = 3708/*N* = 69 and ^b^ *N* = 108/*N* = 9 for cataract surgery and cataract surgery + trabe study groups, respectively.

Abbreviations: DM, diabetes mellitus; PXF, pseudoexfoliation.

### 3.1 Risk of pseudophakic cystoid macular oedema

New‐onset PCME was observed up to 91 days after the surgery, with a mean interval between the surgery and PCME of 47 days (phaco‐alone: 47.9 ± 23.8 days, phaco‐trab: 45.3 ± 23.2 days, *p* = 0.628). PCME rate in the phaco‐alone group was similar (1.04%) to the combined phaco‐trab group (0.35%), *p* = 0.245. In multivariate regression analysis adjusted for age and gender, comparison between the study groups remained comparable (phaco‐alone: OR 0.398, 95% CI 0.099–1.605, *p* = 0.195; phaco‐trab: OR 0.347, 95% CI 0.049–2.477, *p* = 0.291). The risk for PCME was higher among male patients than females (OR 1.324, 95% CI 1.122–1.562, *p* = 0.001) and higher for older individuals (OR 1.007, 95% CI 1.001–1.014, *p* = 0.032).

### 3.2 Risk of posterior capsule opacification

**FIGURE 1:**
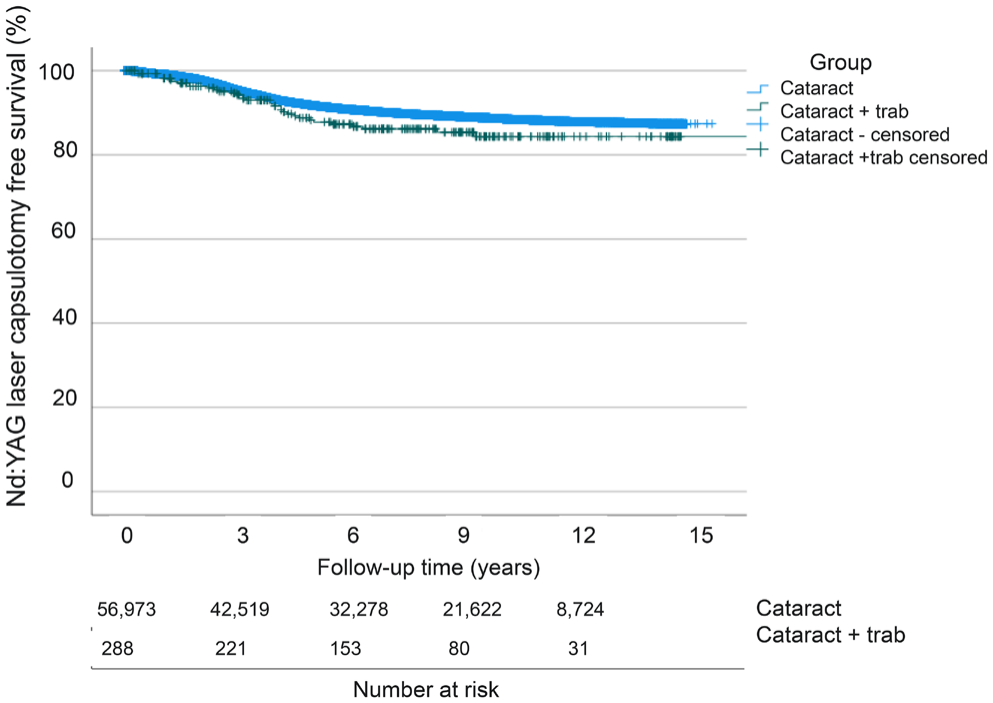
Univariate Nd:YAG laser capsulotomy free survival. Univariate analysis of the cumulative incidence of Nd:YAG laser capsulotomy‐free survival rate (%) in the follow‐up (years) after surgery.

**FIGURE 2:**
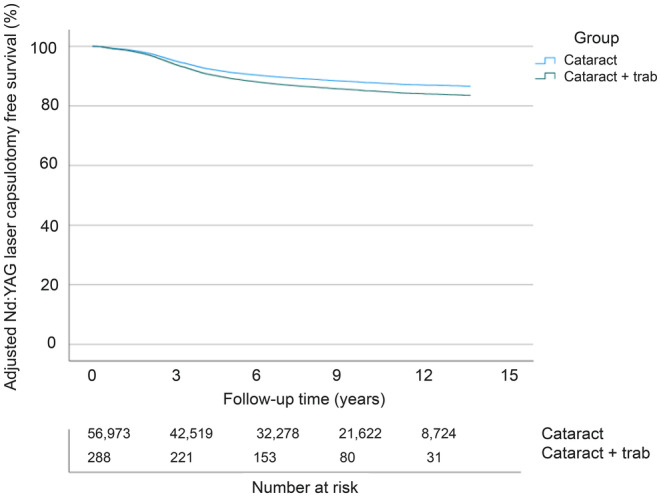
Multivariate Nd:YAG laser capsulotomy free survival. Multivariate analysis – adjusted for age and gender – of the cumulative incidence of Nd:YAG laser capsulotomy‐free survival rate (%) in the follow‐up (years) after surgery.

Nd:YAG laser capsulotomy rates, a surrogate marker for clinically significant PCO development, between the operated eyes without and with concomitant trabeculectomy were compared by univariate Kaplan‐Meier log‐rank analysis (*p* = 0.067, Figure 1). In Cox regression analysis adjusted for the patient's age and gender, Nd:YAG laser capsulotomy rates remained comparable between the groups (*p* = 0.209, Figure 2).

## 4 DISCUSSION

This study investigated the association of a combination of cataract surgery and trabeculectomy with postoperative PCME and PCO using a database of over 55 000 patients who underwent phacoemulsification. We found no statistically significant difference in PCME or PCO between the groups. Population growth, ageing and recent advancements in cataract surgery resulted in a significant increase in cataract surgery rates. Moreover, these factors increase the frequency of concurrent, operable age‐related eye disorders, such as co‐existent vision‐threatening cataracts and glaucoma with difficult‐to‐control IOP. In such cases, combined phaco‐trabeculectomy surgery may be a reasonable approach. Several studies compared the clinical outcomes of phacoemulsification with and without concomitant trabeculectomy. However, these were relatively small studies whose primary outcomes were IOP reduction, a decrease in anti‐glaucoma medications or postoperative BCVA improvement (Anders, 1997; Chelerkar et al., 2018; Chung et al., 2018; Dhalla et al., 2017; El Sayed et al., 2019; Ghadamzadeh et al., 2022; Hansapinyo et al., 2020; Hou et al., 2015; Lazcano‐Gómez et al., 2014; Liaska et al., 2014; Paul et al., 2014; Rhiu et al., 2010; Senthil et al., 2022; Storr‐Paulsen et al., 1998; Tham et al., 2009; Ventura‐Abreu et al., 2021). Indeed, most studies found combined phaco‐trab surgery to be significantly more effective in lowering IOP than cataract surgery alone.

Combined phaco‐trab surgery could potentially lead to postoperative complications, as the rapid reduction in IOP after glaucoma filtering surgery may increase postoperative ocular blood flow, especially in the central retinal area and possibly even the choroid, resulting in central macular thickening and retinal changes (Nilforushan et al., 2022). In addition, the iridectomy and the sclerotomy in trabeculectomy surgery may be sufficiently traumatic to the blood–ocular barrier to increase its permeability. Disruptions of the barrier alter the aqueous composition and possibly lead to an immune‐mediated inflammatory response in susceptible individuals (Leaming, 2003). The additional surgical steps performed while combining a trabeculectomy with phaco surgery have the potential to lead to increased inflammation compared to phaco alone, as was observed by Zarnowski et al. (1998), who concluded that the amount of inflammation was the highest after phaco‐trab compared to phaco‐alone or trab‐alone.

In our previous RCTs on routine cataract surgeries, the incidence of PCME varied between 3.0% and 3.7% (Ilveskoski et al., 2020; Lindholm et al., 2020; Ylinen, Holmström, et al., 2018; Ylinen, Taipale, et al., 2018). In a real‐world study by Chu et al. (2016), the PCME rate within 3 months after cataract surgery was 1.17% in the reference cohort. In a study by Lee et al., the PCME rate was greater among eyes with POAG than among those with uneventful cataract surgery. This finding was associated with perioperative prostaglandin analogue use (Lee et al., 2017).

Table S1 depicts the reported rate of PCME in combined phaco‐trab versus. phaco alone. Both studies by Ventura‐Abreu et al. and Ghadamzadeh et al. showed that phaco‐trab subjects had a higher rate of PCME compared to phaco‐alone subjects (3.7%–4.8% vs. 0%). However, no *p*‐value was reported for these comparisons, and the overall sample size was low (phaco‐trab: 21–27 eyes; phaco alone: 21–25 eyes) (Ghadamzadeh et al., 2022; Ventura‐Abreu et al., 2021). We did not find a significant difference between the groups regarding the risk of postoperative PCME in our larger study (phaco‐trab: 288 eyes; phaco alone: 56973 eyes). To note, the lack of an unambiguous definition or diagnostic criteria for PCME is a generally known problem (Chu et al., 2016; Grzybowski et al., 2016; Kessel et al., 2014; Taipale et al., 2019; Wielders et al., 2019). Deciding on a uniform definition for PCME to be used worldwide would help compare PCME rates between different studies.

Hecht et al. have shown that PCO rates four years after cataract surgery, estimated by the number of Nd:YAG laser capsulotomies, are 10.2% of patients (Hecht et al., 2020). Shin et al. (1998) showed in their study that the rates of PCO, estimated by Nd:YAG laser capsulotomies, were 29% and 30% for patients undergoing combined phaco‐trab surgery and phacoemulsification alone, respectively. We also assessed PCO rates by Nd:YAG laser capsulotomy and found comparable rates of Nd:YAG laser capsulotomy‐free survival between patients who underwent cataract extraction with and without trabeculectomy, with a mean follow‐up of 6.9 ± 4.2 and 6.5 ± 4.0 years, respectively. Results remained statistically insignificant even after adjusting for age and gender. Moreover, the use of adjunctive MMC in combined trabeculectomy and cataract surgery has previously shown potential for reducing the number of lens epithelial cells in the mitotic phase of the cell cycle and a beneficial effect on the prevention of PCO (Shin et al., 1998). This may imply that concomitant trabeculotomy can be safely performed during cataract surgery without increasing the risk of PCO development.

There are several limitations to the current study. First, the study's retrospective nature makes it inherently prone to selection bias, as there is usually a delay between surgery (phaco or phaco‐trabeculectomy) and the development of symptomatic PCO. Patients referred from elsewhere for glaucoma surgery are more likely to go back to their local referral source for simple clinic reviews and procedures such as posterior capsulotomy. Patients who move back to their local area and have YAG laser capsulotomy will be lost to follow‐up and analysis. However, we tried to overcome this bias by including as many patients as possible. In addition, due to the study retrospective design, several confounders could not be controlled for (glaucoma type, Mitomycin‐C application time and post‐operative procedures such as suture lysis, bleb needling or antimetabolite injection), which might affect the results. Specifically, we did not analyse IOL design, which is closely related to PCO. It could be that glaucoma surgeons may choose to use specific IOL models that may not be used by surgeons who performed only cataract surgery. In addition, in large retrospective studies by us and others, Nd:YAG laser capsulotomy rates (a surrogate marker for clinically relevant PCO) after routine cataract cases varied greatly based on the IOLs used in the clinical practice. In a study by Ursell et al., the 3‐year incidence of Nd:YAG capsulotomy was between 2.4% and 12.6% based on the IOL model (Ursell et al., 2020). Belda et al. (2022) reported a 3‐year incidence of Nd:YAG capsulotomy between 5.0% and 31.1% based on the IOL model. In another study, the 3‐year incidence of Nd:YAG capsulotomy after cataract surgery varied between 11.4% and 18.6% based on the IOL model (Leydolt et al., 2020). The 5‐year cumulative incidence of Nd:YAG capsulotomy after cataract surgery was 13.2%, and it ranged between 9.6% and 18.1% according to the IOL model (Lindholm et al., 2019). Second, as the incidence of PCME and PCO is not exceptionally high in the first place, a significant difference may not be found due to a small sample size. Moreover, PCME cases were detected by diagnosis codes in the EMR system, which may result in both over‐ or under‐diagnosis of the condition. Third, the protocol for postoperative follow‐up for cataract surgery may be shorter (up to 2–4 weeks) than for phaco‐trab (months or even years). However, despite a more frequent follow‐up for the phaco‐trab cohort compared to phaco‐alone, no increased incidence of PCME or PCO was observed in the phaco‐trab group. Forth, there is a disparity in sample size between the two groups, as phaco‐trab is a relatively uncommon procedure compared to phaco alone. Lastly, the study is based on data from a single centre with a homogenous population, which may lower the results' external validity.

To conclude, our large registry‐based study showed no significant difference between patients who underwent phacoemulsification cataract surgery alone compared to a combined phaco‐trabeculectomy procedure regarding PCME and Nd:YAG laser capsulotomy rates.

## CONFLICT OF INTEREST STATEMENT

No conflicting relationships exist for any author.

## Supporting information


Table S1.
