# Supplementary material for: Pseudophakic cystoid macular oedema and posterior capsular opacification rates after combined phaco‐trabeculectomy vs. phaco alone
Source: Acta Ophthalmol. 2024 Oct 11;103(1):115–20. doi: 10.1111/aos.16766 (PMC11704849; doi:10.1111/aos.16766)
Supplement: Supplementary file 1 — Table S1. [file AOS-103-115-s001.docx]

Supplemental Table 1. Literature review of PCME and PCO rates following combined phaco-trab vs. phaco alone.

|  |  |  |  |  |  | PCME | | | PCO | | |
| --- | --- | --- | --- | --- | --- | --- | --- | --- | --- | --- | --- |
| Author | Year | Country | n. Phaco-Trab | n. Phaco-alone | Follow-up length (months) | Phaco | Phaco-Trab | P | Phaco | Phaco-Trab | P |
| Shin et al.^a^ | 2002 | USA | 100 | 100 | 56.2 ± 19.0 |  |  |  | 30.0% | 29.0% | 0.877 |
| Tham et al.^a^ | 2010 | China | 61 |  | 24.0 ± 0.0 |  |  |  | 1.6% | 1.6% | 0.991 |
| Paul et al. ^b^ | 2014 | India | 114 | 118 | 24.0 ± 0.0 |  |  |  | 0% | 2.6% | N.A |
| Hansapinyo et al. ^b^ | 2020 | China | 61 | 62 | 85.3 ± 18.4 |  |  |  | 9.7% | 6.6% | 0.526 |
| Ventura-Abreu et al.^b^ | 2021 | Spain | 21 | 21 | 12.0 ± 0.0 | 0% | 4.8% | N.A |  |  |  |
| Ghadamzadeh et al. ^b^ | 2022 | Iran | 27 | 25 | 6.0 ± 0.0 | 0% | 3.7% | N.A |  |  |  |
| PCME; pseudophakic cystoid macular edema, PCO; posterior capsule opacification. Study design: ^a^Retrospective; ^b^Prospective | | | | | | | | | | | |
